# Reversible Inactivation of the Bed Nucleus of the Stria Terminalis Prevents Reinstatement But Not Renewal of Extinguished Fear^1^,^2^,^3^

**DOI:** 10.1523/ENEURO.0037-15.2015

**Published:** 2015-07-03

**Authors:** Travis D. Goode, Janice J. Kim, Stephen Maren

**Affiliations:** Institute for Neuroscience and Department of Psychology, Texas A&M University, College Station, Texas 77843

**Keywords:** BNST, extinction, fear, muscimol, reinstatement, renewal

## Abstract

The extinction of conditioned fear is labile. For example, fear to an extinguished conditioned stimulus (CS) returns after presentation of an aversive stimulus (“reinstatement”) or a change in context (“renewal”). Substantial research implicates the bed nucleus of the stria terminalis (BNST) in the stress-induced relapse of extinguished behaviors, such as in instrumental drug seeking, but its role in the relapse of extinguished fear responses is not clear. Here, we explored the role of the BNST in both the reinstatement and renewal of fear, two forms of relapse that are differentially triggered by stress. In Experiment 1, rats received pairings of an auditory CS and footshock unconditioned stimulus (US) followed by an extinction procedure. After extinction, rats received an unsignaled US to reinstate fear to the extinguished CS. Twenty-four hours later, they were infused with either muscimol or vehicle into the BNST immediately prior to a CS retrieval test. In Experiment 2, rats were conditioned and extinguished in two distinct contexts. Twenty-four hours after extinction, the rats were infused with muscimol, NBQX, or vehicle immediately prior to a CS retrieval test in either the extinction context or a different (but familiar) context. In both experiments, freezing behavior served as the index of conditioned fear. The results revealed that BNST inactivation prevented reinstatement (Experiment 1), but not renewal (Experiment 2), of conditioned freezing to the extinguished CS. Hence, the BNST is critical for the reinstatement of extinguished fear in an aversive context, but not for the contextual retrieval processes that mediate fear renewal.

## Significance Statement

Relapse of extinguished fear is a major challenge to clinical treatments of fear-related anxiety disorders (e.g., exposure therapy). Pavlovian fear conditioning and extinction are important models for understanding the behavioral and brain mechanisms underlying fear relapse. Here we explore the role of the bed nucleus of the stria terminalis (BNST) in two different forms of fear relapse in rats: “reinstatement” and “renewal.” We find that reversible inactivation of the BNST prevents the reinstatement, but not the renewal, of extinguished fear. This reveals a dissociation in the role of the BNST in different forms of relapse, a finding that will serve to enhance the selectivity of neural interventions for anxiety disorders.

## Introduction

Fear relapse plagues clinical interventions for fear-related anxiety disorders (Hooley, 2007; Boschen et al., 2009; Vervliet et al., 2013b). Various factors—such as the nature of the therapeutic intervention, duration of time since treatment, and intervening stress—have been shown to be important in determining the degree of retention of extinguished fear in humans and other animals (Kehoe and Macrae, 1997; Bouton, 2002, 2014; Myers and Davis, 2002; Bouton et al., 2006; Hermans et al., 2006; Fitzgerald et al., 2014; Goode and Maren, 2014; Luck and Lipp, 2015). Pavlovian fear conditioning and extinction in rodents provides a clinically relevant model to explore the behavioral and brain mechanisms of relapse. Specifically, fear conditioning in rats is a behavioral procedure through which subjects experience concomitant pairings of a neutral conditioned stimulus (CS), such as a tone, with an aversive unconditioned stimulus (US), such as a footshock (Rescorla, 1988a,b). After fear conditioning, presentation of the CS alone comes to elicit conditioned fear responses (CRs), including freezing behavior (Fendt and Fanselow, 1999; LeDoux, 2000; Maren, 2001). Fear CRs also occur in the place or “context” in which fear conditioning was experienced (Bouton and King, 1983; Maren et al., 2013).

After conditioning, repeated presentations of the CS in the absence of footshock lead to the extinction of fear (Pavlov, 1927; Chang et al., 2009). It is widely believed that research on fear extinction in rodents can enhance our understanding of exposure therapy in humans (Barad, 2005; Milad et al., 2006, 2014; Morrison and Ressler, 2014). Extensive research indicates that extinction training does not necessarily erase fear memory; rather, it results in a new “inhibitory” memory that limits the expression of the fear (Konorski, 1967; Bouton, 2004; Maren, 2011). Consequently, extinction memories are susceptible to relapse. Two forms of fear relapse have received considerable attention over the years: “reinstatement” and “renewal.” Reinstatement of fear occurs when an aversive, unsignaled US is experienced prior to presentation of the extinguished CS (Rescorla and Heth, 1975; Bouton and Bolles, 1979). Reinstatement is most robust in contexts in which reinstating shocks are delivered, although it can also occur in contexts never paired with shock (Westbrook et al., 2002; see also Morris et al., 2005a; Halladay et al., 2012; Goode et al., 2015). This suggests that reinstatement can be mediated by either direct context–US associations (Bouton and King, 1983; Bouton et al., 2006) or though stress states that generalize across contexts (Haroutunian and Riccio, 1977; Morris et al., 2005b; Deschaux et al., 2013). Fear renewal, on the other hand, occurs when a CS is presented outside of its extinction context (Bouton and Bolles, 1979; Polack et al., 2013; Vervliet et al., 2013a). Importantly, renewal does not require that the animal experience the US after extinction. Indeed, direct context–US associations do not mediate renewal (Bouton and Ricker, 1994; Harris et al., 2000; Corcoran and Maren, 2004).

The different roles that context plays in reinstatement and renewal suggest that distinct neural circuits mediate them. One brain area that has been implicated in reinstatement is the bed nucleus of the stria terminalis (BNST). In particular, BNST lesions impair the shock-induced reinstatement of fear (Waddell et al., 2006; see also Waddell et al., 2008). Sustained fear responses to conditioned contexts (Sullivan et al., 2004) and long-duration CSs (Waddell et al., 2006) also appear to rely on the BNST (also, see Walker and Davis, 1997). Conversely, BNST manipulations do not affect fear to short-duration CSs paired with shock (LeDoux et al., 1988; Sullivan et al., 2004; Waddell et al., 2006; Zimmerman and Maren, 2011). Coinciding with this evidence, BNST circuitry is also involved in the stress-induced reinstatement of drug seeking. For example, antagonism of corticotropin-releasing factor receptors within the BNST blocks the reinstatement of cocaine seeking after footshock exposure (Erb and Stewart, 1999). Similarly, pharmacological inactivation of the BNST prevents the stress-induced reinstatement of cocaine seeking following systemic administration of the anxiogenic drug yohimbine (Buffalari and See, 2011). Collectively, these data suggest that the BNST may have a selective role in forms of relapse that depend on stress and/or contextual fear, such as in reinstatement. To explore this question, we examined the consequences of reversibly inactivating the BNST in the expression of both the reinstatement and renewal of fear after extinction in rats. We hypothesized that BNST inactivation would attenuate the reinstatement, but not the renewal, of extinguished fear.

## Materials and Methods

### Subjects

All subjects were adult (200-250 g) male Long−Evans (Blue Spruce) rats from Harlan Laboratories. Upon arrival, rats were individually housed in clear plastic cages on a rotating cage rack (Animal Care Systems). Group assignments for behavioral training were randomized for cage position on the racks. Rats were given free access to standard rodent chow and water. Sawdust served as bedding for the rats (bedding was changed once a week). Behavioral experiments took place on different days from the days that cages were changed. Rats were kept on a fixed light/dark cycle, with rats experiencing 14 h of light (starting at 7:00 A.M.) followed by 10 h of darkness each day. All handling, surgeries, and behavioral testing occurred during the light hours of the light/dark cycle. The experimenters handled each rat for 1 min/d for 5 d prior to the start of surgeries. Additionally, rats were habituated to the infusion procedures and to the infusion room prior to behavioral training. The Texas A&M University Institutional Animal Care and Use Committee approved all experimental procedures.

### Surgery

Rats were anesthetized with an intraperitoneal injection of ketamine (100 mg/kg) and xylazine (10 mg/kg), and were treated with atropine methyl nitrate (0.4 mg/kg, i.p.). After the induction of anesthesia, the head of each rat was shaved, and the rats were placed in a stereotaxic frame (David Kopf Instruments). The scalp was incised, and the skull was leveled, with bregma and lambda in the same horizontal plane. Small holes were drilled in the skull and steel guide cannulae (26 gauge, 8 mm; Small Parts) were lowered into the BNST (0 mm anteroposterior to bregma, ±2.7 mm mediolateral, and −5.9 mm ventral to dura). Guide cannulae were angled at 10° to limit penetration of the lateral ventricles. Three stainless steel screws were affixed to the skull, and the entire skull surface was covered with dental cement to secure the cannulae to the skull. Stainless steel obturators (30 gauge, 9 mm; Small Parts) were placed inside each cannula and changed every 2 d prior to behavioral testing. Rats were given a single bacon-flavored Rimadyl tablet (2 mg/tablet; Bio-Serv) following surgery. The rats were allowed at least 1 week to recover from surgery before behavioral testing.

### Behavioral apparatus

Behavioral testing was conducted in two distinct rooms within the laboratory (“Wellborn” and “University”). Each testing room contained eight identical conditioning chambers (Med Associates) fabricated with aluminum (sidewalls) and Plexiglas (rear wall, ceiling, and front cage door) walls (30 × 24 × 21 cm). The conditioning chambers were housed in external sound-attenuating cabinets. The floor of each chamber consisted of 19 stainless steel rods (4 mm in diameter); each rod was spaced 1.5 cm apart (center to center). Each chamber was equipped with a speaker to provide the CS. As described for each context (see below), a 15 W house light provided ambient lighting and a cabinet fan provided background noise for each chamber (∼70 dB). The grid floors of each chamber were connected to a shock source and a solid-state grid scrambler to deliver the footshock US (Med Associates). Each chamber rested on a load-cell platform that detected chamber displacement in response to the movement of each animal. Load-cell activity values (range, −10 to +10 V) were acquired during all behavioral phases and digitized at 5 Hz with Threshold Activity Software (Med Associates). Load-cell output was transformed off-line to values ranging from 0 to 100 (higher values indicate more displacement of the cage). A bout of freezing was scored if the absolute values of load-cell activity were ≤10 for ≥1 s (Maren, 1998). The number of 1 s bins of freezing was divided by the total number of bins in each observation period (typically, a 30 s or 1 min period after each trial) to yield the percentage of time each animal was freezing.

Distinct contexts were created through the use of different odors and visual cues. For Experiment 1, conditioning and extinction occurred in Context A; these chambers were located in the Wellborn test room in the laboratory. For Context A, the cage walls were wiped with acetic acid (1.5%) and a small volume was placed in the trays underneath the grid floor. The houselights were extinguished, but the overhead fluorescent room lights were illuminated. Rats were transported to Context A in white containers, and the cabinet doors enclosing the conditioning chambers were open during testing. The reinstatement sessions occurred in Context B; these chambers were located in the University test room in the laboratory. For Context B, ammonium hydroxide (1%) was used to wipe the cage walls and a small volume was placed in the trays underneath the grid floors. The overhead fluorescent room lights remained off (red room lights provided overhead illumination); the houselights within each testing chamber were illuminated. Rats were transported to Context B in black containers, and the cabinet doors enclosing the conditioning chambers were closed during testing. Cabinet fans were turned on for both Context A and B in Experiment 1. For Experiment 2, conditioning was conducted in Context A as described above, whereas extinction and renewal testing used Contexts B and C (per group assignments; cabinet fans were turned off for Contexts B and C in Experiment 2). For Context C (Wellborn room), ethanol (70%) was used to wipe the cage walls and a small volume was placed in the trays beneath the grid floor. The houselights were illuminated, and the overhead lights were extinguished; a thin black Plexiglas sheet covered the grid floor for Context C. Cabinet doors enclosing the chambers were open during testing in Context C. Rats were transported to Context C in white 5 gallon buckets; a layer of sawdust was placed in each bucket and changed out for each squad of animals.

### Behavioral procedures

#### Experiment 1: effects of BNST inactivation on the expression of reinstatement

**Figure 1.**
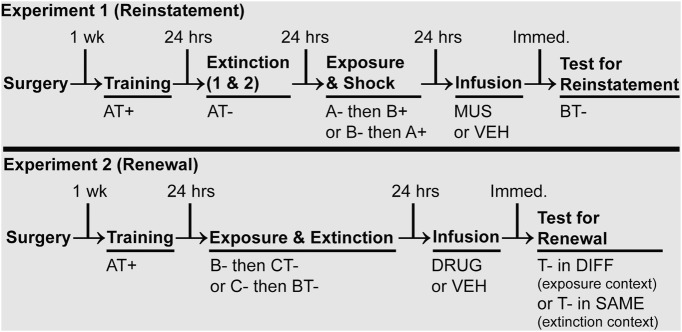
Experimental designs are read from left to right. Each phase of behavior is separated by 24 h; however, infusions occurred immediately prior to testing in both Experiments 1 and 2. A, B, C, experimental contexts; T, tone CS; +, US; −, no US.

An illustration of the behavioral paradigm for Experiment 1 is shown in Figure 1. Prior to behavioral testing, 32 rats were randomly assigned to groups that would receive intracranial infusions of either muscimol (MUS; a selective GABA_A_ receptor agonist; *n* = 16) or vehicle (VEH; physiological saline; *n* = 16) prior to retrieval testing. MUS rats received a total of 0.3 μg of muscimol (1.0 μg/μl in 0.3 µl) per hemisphere. VEH rats were infused with 0.3 μl of physiological saline per hemisphere. All infusions occurred over 1 min at a rate of 0.3 μl/min. Within each drug condition, rats were also randomly assigned to receive reinstatement shock in either the test context (Context B) or the extinction context (Context A). Rats did not differ at test based on the context in which the reinstatement shock was delivered (*F* values < 1); therefore, we collapsed rats across this condition.

 On Day 1, rats were transported to Context A for fear conditioning. Three minutes after placement in the chambers, rats received five auditory CSs (10 s, 2 kHz, 80 dB)–footshock US (2 s, 1 mA) pairings [US onset occurred upon CS offset; 70 s intertrial intervals (ITIs)]. After the final conditioning trial, rats remained in the chambers for 1 min before being returned to their home cages. Twenty-four hours later, rats underwent the first of two extinction sessions. During these sessions, they were returned to the conditioning context (Context A), and, after 3 min, rats were presented with 45 CS-alone trials (40 s ITIs). After the final CS presentation, the rats remained in the chambers for 3 min before being returned to their home cages. The second extinction session was identical to the first and occurred on the following day. Twenty-four hours after the final extinction session, rats underwent a reinstating shock session. First, rats were exposed to either Context A or B for 4 min in the absence of the CS or US; rats were returned to their home cages after this experience. Two hours later, rats were brought back to the laboratory and were placed in the other context (A or B; per group assignments) for a reinstating shock in that context. For the reinstating shock session, rats received a single, unsignaled footshock (1 s, 0.4 mA) after 3 min in chambers. Rats remained in the chambers for 1 min after shock offset. Last, on Day 5, and immediately prior to retrieval testing, the rats were infused with muscimol or vehicle and transported to Context B to assess fear to the extinguished CS. Ten minutes after placement in Context B, the rats received five CS-only presentations (40 s ITIs). Rats remained in the testing chambers for 3 min following the final CS presentation.

#### Experiment 2: effects of BNST inactivation on the expression of renewal

Refer to Figure 1 for an illustration of the behavioral paradigm used for Experiment 2. Seventy-six rats were randomly assigned to drug (DRUG or VEH) and testing [different (DIFF) or SAME] conditions. Immediately prior to renewal testing, DRUG rats were infused with either 0.3 μg of muscimol (1.0 μg/μl in 0.3 µl) per hemisphere (identical to Experiment 1) or 3.0 µg of 2,3-dihydroxy-6-nitro-7-sulfonyl-benzo[*f*]quinoxaline (NBQX; 10.0 μg/μl in 0.3 µl) per hemisphere. NBQX is a potent AMPA receptor and kainate receptor antagonist. We found no difference in the effects of NBQX or muscimol on conditional freezing at test (*F* values < 0.1); therefore, we collapsed DRUG rats across this condition. Rats assigned to receive VEH were infused with 0.3 μl of physiological saline. As in Experiment 1, all infusions were delivered at 0.3 μl/min for 1 min. Rats assigned to the SAME condition were tested to the extinguished CS in the extinction context, whereas rats assigned to the DIFF condition experienced the extinguished CS outside of the extinction context (but in a familiar context).

On Day 1, all rats were conditioned with five CS–US pairings (CS: 10 s, 2 kHz, 80 dB auditory tone; US: 2 s, 1 mA footshock) in Context A (the procedure was identical to Experiment 1). Twenty-four hours later (Day 2), rats were first exposed for 35 min to the context (either Context B or Context C) that was not hosting extinction; this ensured that exposure to all contexts was counterbalanced. Three hours later, the rats were extinguished in the alternate context (either Context B or Context C; counterbalanced by group). Three minutes after placement in the extinction context, the rats received 45 CS-only presentations (40 s ITIs); the rats remained in the chambers for 3 min after the final CS presentation. Twenty-four hours later, and immediately prior to renewal testing, rats were infused with either drug (muscimol or NBQX) or VEH and transported to the appropriate test context (which was either the same as or different from the extinction context). Responding at the test was not affected by whether the renewal context was B or C (*F* values < 0.1); the data are collapsed across this condition. Three minutes after placement in the test context, rats received five CS-only presentations (40 s ITIs). Rats remained in the testing chamber for 3 min after the final CS-only presentation.

### Intracranial infusions

Rats were transported in 5-gallon buckets to a procedure room within the colony for drug infusions. The obturators were removed from the guide cannulae and stainless steel injection needles (33 gauge, 9 mm; extending 1 mm beyond the end of the guide) were inserted. Each injector (Small Parts) was attached to polyethylene tubing (PE-20; Braintree Scientific), which in turn was connected to a gastight 10 μl syringe (Hamilton, Co.). Syringes were mounted in an infusion pump (KD Scientific). After insertion of the injectors, the rats were returned to the buckets where they remained unrestrained during the infusion procedure. After the infusion, the injection needles remained in the guide cannulae for 1 min before being removed; clean obturators were inserted into the guides, and the rats were transported to the conditioning chambers.

### Histological procedures

**Figure 2:**
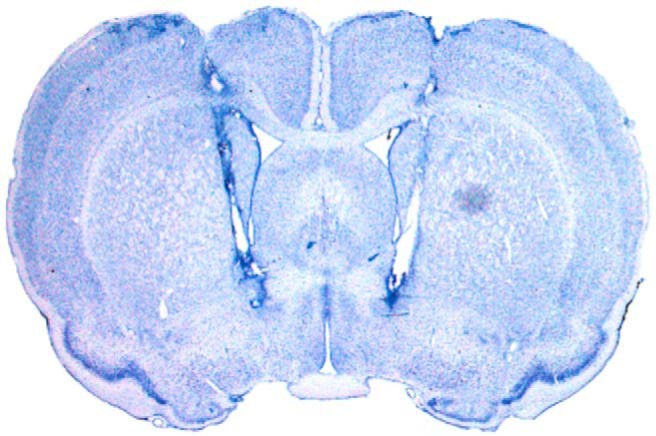
Representative photomicrograph of a thionin-stained coronal section (40 μm) from the brain of a rat with injector tips terminating within the bed nucleus of the stria terminalis.

Within 1 week after the final retention test, the rats were overdosed with sodium pentobarbital (Fatal Plus; 100 mg/ml, 0.5 ml, i.p.) and perfused. Transcardial perfusions were performed with physiological saline followed by 10% formalin solution. Brains were removed from the skull and stored in 10% formalin for 24 h at 4° C followed by 30% sucrose-formalin for at least 3 d before sectioning. Brain tissue was flash frozen with dry ice and sectioned at 40 μm on a cryostat (Leica Microsystems) at −20° C. Sections were wet mounted to microscope slides and stained with 0.25% thionin to identify cannula tracts and to localize injection sites in the tissue. Photomicrographs of the sections (10× magnification) were captured and digitized using a Leica MZFLIII microscope. Figure 2 shows a representative coronal section from a rat with injector tips localized to the BNST.

### Data analyses

Freezing served as the index of fear for all behavioral analyses. All data were submitted to ANOVA (in-text lowercase superscripts correspond to the analyses in Table 1). *Post hoc* comparisons (Fisher’s protected least significant difference test) on individual group means were calculated after a significant omnibus *F* ratio in the ANOVA; α was set at 0.05. Rats were excluded from the analyses if they failed to extinguish by the final extinction session (mean freezing, >50%) or if mean pre-CS freezing during the retrieval test was >50%. Based on these criteria, 10 rats were excluded from Experiment 2. Unless noted otherwise, freezing data (as a percentage of the total time spent immobile) were analyzed across the trials in Figures 4, 5.

## Results

### Histology

**Figure 3:**
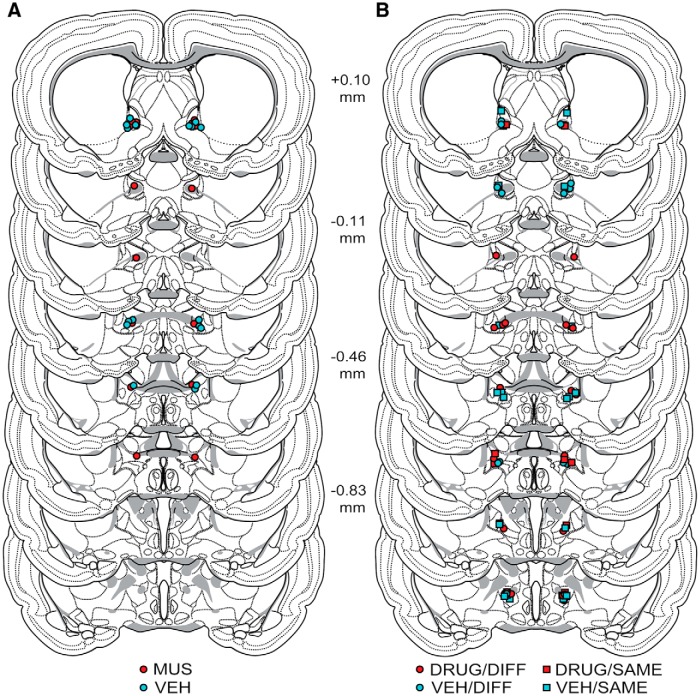
Illustration of cannula placement sites in the bed nucleus of the stria terminalis. Placements are shown for all rats included in the final analyses for Experiment 1 (***A***) and Experiment 2 (***B***). Adapted from Swanson (1998). Distances shown are relative to bregma.

Injection sites within the BNST are illustrated in Figure 3. For Experiment 1 (Fig. 3*A*), 16 injectors terminated within the anterior lateral division of the BNST (which includes the anterolateral area, juxtacapsular nucleus, oval nucleus, and rhomboid nucleus), 4 were localized to the anterior medial BNST (which includes the anterodorsal area and central core of the anterodorsal area), 5 were localized to the anterior ventral BNST (which includes the anteroventral area, dorsolateral nucleus, dorsomedial nucleus, fusiform nucleus, magnocellular nucleus, subcommissural zone, and ventral nucleus), and 9 were located in the anterior commissure within the anterior BNST (Swanson, 1998). This yielded the following groups for the final analyses: MUS, *n* = 7; VEH, *n* = 10. Cannulae missed their targets in 15 animals; these animals (MUS, *n* = 9; VEH, *n* = 6) were analyzed separately to determine whether off-target drug infusions affected reinstatement.

For Experiment 2, 45 animals received bilateral injectors within the BNST. Of these animals, 10 subjects were excluded based on the behavioral criteria described above, yielding the following groups: DRUG/DIFF, *n* = 11; DRUG/SAME, *n* = 6; VEH/DIFF, *n* = 8; and VEH/SAME, *n* = 10. Accordingly, 70 injection sites from 35 animals are illustrated in Figure 3*B*. Thirteen injectors were localized to the anterior lateral division of the BNST, 4 terminated within the anterior medial division, 22 terminated within the anterior ventral division, 5 terminated in the anterior commissure within the anterior BNST, and 26 terminated within the posterior division of the BNST (which includes the interfascicular nucleus, principal nucleus, and transverse nucleus).

### Experiment 1: BNST inactivation prevents reinstatement of fear to an extinguished CS

**Figure 4:**
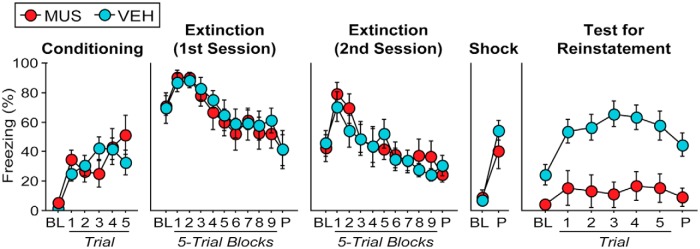
Pharmacological inactivation of the BNST prevents reinstatement (Experiment 1). Conditioning, Mean (±SEM) percentage of freezing during the 3 min baseline (BL) and in the 60 s interstimulus interval following each CS–US pairing. Extinction (First Session), Mean (±SEM) percentage freezing during a 3 min BL and across nine extinction blocks (each block represents average responding during the 30 s post-CS intervals after five extinction trials). The rats remained in the chambers for 150 s after the final CS presentation (P). Extinction (Second Session), Mean (±SEM) percentage freezing for the second day of extinction training (trials are equivalent to the first extinction day). Shock, Mean (±SEM) percentage freezing during the 3 min BL period before unsignaled footshock and during the 1 min postshock period. Test for Reinstatement, Mean (±SEM) percentage freezing during the final 3 min of the BL period immediately prior to CS onset and during five 30 s interstimulus intervals after each test trial; the rats remained in the chambers for 150 s after the final CS.

Rats exhibited reliable fear conditioning to the auditory CS (Fig. 4). This impression was confirmed in an ANOVA by a significant main effect of trial (*F*_(5,75)_ = 9.1; *p* < 0.0001^a^); freezing behavior increased across the conditioning session, and there were no group differences on this measure (*F* values < 1). Over the next 2 d, all rats were extinguished to the CS in Context A. During the first extinction session (Fig. 4), there was a significant main effect of trial (*F*_(10,150)_ = 10.6; *p* < 0.0001^b^) as freezing behavior decreased over the course of the extinction session; there were no group differences in extinction rate or magnitude (*F* values < 1). During the second extinction session (Fig. 4), there again was a significant main effect of trial (*F*_(10,150)_ = 7.9; *p* < 0.0001^c^), reflecting decreases in freezing behavior over the course of the session; again, there were no group differences in extinction rate or magnitude (*F* values < 1). On Day 4, all rats received an unsignaled footshock to reinstate fear to the extinguished CS (Fig. 4). Freezing behavior reliably increased after footshock. This impression was confirmed in an ANOVA that revealed a significant main effect of trial for freezing across the preshock and postshock periods (*F*_(1,15)_ = 49.5; *p* < 0.0001^d^). Shock-induced increases in fear on Day 4 were similar across drug and context conditions (*F* values < 2).

**Table 1:** Statistical table

In-text letter	Data structure	Type of test	Power
a	Normal distribution	One-way repeated-measures ANOVA (conditioning trials)	1.000
b	Normal distribution	One-way repeated-measures ANOVA [extinction trials (Day 2)]	1.000
c	Normal distribution	One-way repeated-measures ANOVA [extinction trials (Day 3)]	1.000
d	Normal distribution	One-way repeated-measures ANOVA (reinstating shock trials)	1.000
e	Normal distribution	One-way repeated-measures ANOVA (infusion group across baseline at test)	0.968
f	Normal distribution	One-way repeated-measures ANOVA (infusion group across testing)	0.957
g	Normal distribution	One-way repeated-measures ANOVA (test trials)	0.993
h	Normal distribution	One-way repeated-measures ANOVA (final extinction block vs mean responding at test for VEH animals)	0.935
i	Normal distribution	One-way repeated-measures ANOVA (conditioning trials)	1.000
j	Normal distribution	One-way repeated-measures ANOVA (extinction trials)	1.000
k	Normal distribution	One-way repeated-measures ANOVA (renewal group across testing)	0.951
l	Normal distribution	Two-way repeated-measures ANOVA (testing trials × renewal group)	0.858
m	Normal distribution	One-way repeated-measures ANOVA (final extinction block vs mean responding at test for DIFF animals)	0.977

Twenty-four hours after the reinstatement shock, rats were infused with muscimol or vehicle into the BNST and immediately placed in Context B for a CS retrieval test. During the 10 min baseline prior to the first CS presentation, vehicle-treated rats exhibited significantly greater levels of freezing than muscimol-treated animals (VEH, 31.108 ± 5.644%; MUS, 3.968 ± 1.409%). This was confirmed in the ANOVA by a significant main effect of drug across baseline freezing (*F*_(1,50)_ = 15.423; *p* = 0.0013^e^). In addition, and as shown in Figure 4, VEH-treated rats exhibited significantly greater levels of fear to the extinguished CS than MUS-treated animals. This impression was confirmed in the ANOVA by a main effect of drug across all testing trials (*F*_(1,90)_ = 14.446; *p* = 0.0017^f^). There was a significant main effect of trial (*F*_(6,90)_ = 5.737; *p* < 0.0001^g^) insofar as freezing behavior increased on average after presentation of the CS. Freezing to the CS during the retrieval test in vehicle-treated rats was significantly greater than during the final block of extinction, indicating successful reinstatement of extinguished fear (*F*_(1,9)_ = 14.607; *p* = 0.0041^h^). Importantly, reinstatement impairments were obtained only in rats with cannula placements in the BNST. Muscimol infusion in rats with off-target placements that missed the BNST exhibited normal reinstatement and did not differ from controls during either the baseline or CS periods (*F* values < 0.5). Overall, these data reveal that BNST inactivation reduced both contextual freezing and the reinstatement of fear to an extinguished CS.

### Experiment 2: BNST inactivation does not alter the expression of fear renewal

**Figure 5:**
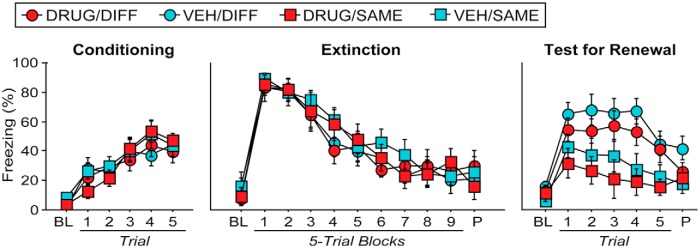
Pharmacological inactivation of the BNST does not prevent renewal (Experiment 2). Conditioning, Mean percentage of freezing during the 3 min baseline (BL) and in the 60 s interstimulus interval following each CS–US pairing. Extinction, Mean (±SEM) percentage freezing during a 3 min BL and across nine extinction blocks. Each block represents the average responding during the 30 s post-CS intervals after five extinction trials. The rats remained in the chambers for 150 s after the final CS presentation (P). Test for Renewal, Mean (±SEM) percentage freezing during the 3 min baseline period immediately prior to CS onset and during five 30 s interstimulus intervals after each test trial; the rats remained in the chambers for 150 s after the final CS.

As shown in Figure 5, rats exhibited reliable fear conditioning. This impression was confirmed in the ANOVA by a significant main effect of trial (*F*_(5,155)_ = 31. 2; *p* < 0.0001^i^); freezing behavior increased over the course of conditioning, and the groups did not differ from one another (*F* values < 1). Twenty-four hours later, rats significantly reduced their fear across extinction trials (Fig. 5; main effect of trial, *F*_(10,310)_ = 50.6; *p* < 0.0001^j^). Extinction of fear on Day 2 was similar across group assignments (*F* values < 1).

On Day 3, rats were infused with either drug or vehicle immediately before receiving a retrieval test in the extinction context (SAME) or in the context in which extinction did not occur (DIFF). As indicated in Figure 5, both VEH- and DRUG-treated rats exhibited robust fear renewal in the DIFF context relative to the low levels of freezing expressed by rats in the SAME context. BNST inactivation did not attenuate the renewal of fear to the extinguished CS. This impression was confirmed in the ANOVA by a main effect of context (*F*_(1,186)_ = 12.843; *p* = 0.0011^k^) such that rats in the DIFF condition exhibited significantly more freezing across test trials than those in the SAME condition (regardless of drug condition). A significant trial × context interaction (*F*_(6,186)_ = 2.647; *p* = 0.0173^l^) indicated that DIFF rats exhibited greater freezing after CS onset compared to SAME rats. Moreover, DIFF rats (but not SAME rats; *F* values < 0.5) exhibited significantly more freezing across test trials relative to the final block of extinction (*F*_(1,18)_ = 16.005; *p* = 0.0008^m^), indicating robust renewal of fear. Hence, BNST inactivation did not impair either the renewal or expression of fear to an extinguished auditory CS.

## Discussion

The current study reveals the novel finding that the BNST plays a specific role in the shock-induced reinstatement of extinguished fear; BNST inactivation did not affect the renewal of extinguished fear that accompanies a change in context. Deficits in the reinstatement of extinguished fear were paralleled by reductions in the expression of contextual freezing after BNST inactivation. These results are consistent with an earlier report (Waddell et al., 2006) revealing that neurotoxic lesions of the BNST impair the shock-induced reinstatement of extinguished fear. Additionally, our work parallels findings revealing that the BNST is necessary for shock-induced reinstatement of extinguished drug-seeking behavior (Erb and Stewart, 1999; Erb et al., 2001; see also Leri et al., 2002; Buffalari and See, 2011). Collectively, these data suggest that the BNST has a critical role in the relapse of extinguished behaviors caused by the experience of aversive stimuli (for review, see Smith and Aston-Jones, 2008; Silberman and Winder, 2013; Stamatakis et al., 2014).

Although BNST inactivation impaired fear reinstatement, it did not affect fear renewal despite the fact that the extent of relapse was similar between experiments. This reveals that deficits in reinstatement are not due to impairments in the expression of freezing per se. Indeed, this pattern of results is consistent with other reports indicating that the BNST has a selective role in the expression of fear to contextual compared to discrete CSs (LeDoux et al., 1988; Walker and Davis, 1997; Sullivan et al., 2004; Waddell et al., 2006, 2008; Zimmerman and Maren, 2011; Sink et al., 2013; see also Duvarci et al., 2009; Haufler et al., 2013). Importantly, pretraining lesions of the BNST do not disrupt the acquisition of conditioned fear to discrete CSs (LeDoux et al., 1988; Waddell et al., 2006), nor do post-training lesions of the BNST affect the expression of conditioned fear to discrete CSs (Sullivan et al., 2004). However, BNST lesions attenuate the expression of fear to shock-associated contexts (Sullivan et al., 2004) and attenuate the expression of fear responses to long-duration CSs (i.e., 10 min tones paired with shock; Waddell et al., 2006). Additionally, Sullivan et al. (2004) reported that rats with BNST lesions exhibited blunted corticosterone responding during exposure to a conditioned context (see also Resstel et al., 2008). Hence, it is believed that BNST inactivation prevents reinstatement by reducing the expression of contextual fear, which is thought to be essential for the reinstatement effect (Bouton and Bolles, 1979; Westbrook et al., 2002; Bouton et al., 2006; Waddell et al., 2006).

A key finding in the present study is that BNST inactivation did not affect fear renewal. Unlike reinstatement, however, renewal does not require contextual fear. Indeed, fear renewal can be obtained in contexts that have never hosted shock (e.g., “ABC” or “AAB” renewal; Bouton and Bolles, 1979; Bouton and Ricker, 1994; Harris et al., 2000; Westbrook et al., 2002; Corcoran and Maren, 2004; Holmes and Westbrook, 2013; Jin and Maren, 2015). Renewal also occurs in shock-associated contexts that have themselves undergone extinction and no longer support contextual fear (e.g., “ABA” renewal; Bouton and King, 1983; Vansteenwegen et al., 2005; Effting and Kindt, 2007; Knox et al., 2012; Polack et al., 2013; Holmes and Westbrook, 2014). These findings support the idea that renewal depends not on direct context–US associations, but on a contextual retrieval process that informs the animal of what a CS means in a particular context (Bouton and Bolles, 1979; Bouton and King, 1983; Bouton and Peck, 1989; Holland, 1992; Bouton, 1993; Bouton and Ricker, 1994; Harris et al., 2000; Bouton et al., 2006; Ji and Maren, 2007; Maren et al., 2013; Vervliet et al., 2013b; Delamater and Westbrook, 2014). Importantly, the present results strengthen this view insofar as renewal of fear was immune to BNST inactivation (a manipulation that impairs contextual fear). Considerable work now reveals that this contextual retrieval process depends on a circuit involving the amygdala, hippocampus, and prefrontal cortex (Corcoran and Maren, 2004; Ji and Maren, 2005, 2007; Herry et al., 2008; Knapska and Maren, 2009; Orsini et al., 2011, 2013; Zelikowsky et al., 2012; Maren, 2014; Jin and Maren, 2015).

An important issue that has yet to be resolved is which subregions of the BNST contribute to the effects we have observed in the present study. Indeed, the BNST is heterogeneous in structure, and different subregions within the BNST appear to make unique contributions to fear and anxiety (Walter et al., 1991; Dong et al., 2001a; Dong and Swanson, 2003, 2004a,b; Choi et al., 2007; Jennings et al., 2013; Kim et al., 2013). For example, Kim et al. (2013) showed that photostimulation of the oval nucleus of the BNST resulted in anxiety-related behaviors, while photostimulation of the anterodorsal region of the BNST resulted in anxiolytic behaviors. Jennings et al. (2013) demonstrated that photostimulation of glutamatergic projections of the ventral BNST to the ventral tegmental area (VTA) produced increases in anxiety, whereas photostimulation of GABAergic BNST projections to the VTA was anxiolytic. In the present study, cannula placements terminated primarily within the anterior portion of the BNST (particularly the anterior lateral and anterior ventral divisions of the BNST), though several rats received infusions within the posterior division of the BNST in Experiment 2. The spread of drug likely affected multiple BNST nuclei in these areas. Circuit-selective chemogenetic or optogenetic techniques would help to clarify the specific BNST subregions contributing to fear reinstatement (Sparta et al., 2013).

In humans and other animals, the BNST shares connections with several important emotion-regulating regions in the brain, including the amygdala, dorsal raphe nucleus, hippocampus, hypothalamus, nucleus accumbens, prefrontal cortex, and ventral tegmental area (Swanson and Cowan, 1977; Weller and Smith, 1982; Phelix et al., 1992; Sun and Cassell, 1993; Dong et al., 2001a,b; Dong and Swanson, 2003, 2004a,b; Jalabert et al., 2009; Crestani et al., 2013; Avery et al., 2014; Roman et al., 2014; Krüger et al., 2015). Not surprisingly, the BNST has been implicated in various depression- and anxiety-related behaviors (for review, see Walker and Davis, 2008; Hammack et al., 2009, 2010,2012; Walker et al., 2009; Davis et al., 2010; McElligott et al., 2013; Adhikari, 2014; Kash et al., 2015). Importantly, the BNST modulates hypothalamic–pituitary–adrenal axis activity, including corticosterone release, via its connections with the hypothalamic paraventricular nucleus (Cullinan et al., 1993; Herman et al., 1994; Sullivan et al., 2004; Choi et al., 2007; Crestani et al., 2013). Corticosterone release is correlated with both the acquisition and expression of conditioned fear (Campeau et al., 1997; Pugh et al., 1997; Cordero et al., 1998; Roozendaal et al., 2006; Marchand et al., 2007). Hence, BNST lesions might influence reinstatement by limiting the modulatory effects of corticosterone on fear expression to an extinguished CS. Alternatively, the BNST is positioned to directly influence freezing behavior via its projections to the amygdala and periaqueductal gray (Dong et al., 2001a; Dong and Swanson, 2003, 2004a,b; Fendt et al., 2003; Asok et al., 2013). In this way, the BNST might directly drive reinstatement of fear to an extinguished CS by driving amygdaloid and periaqueductal gray circuits involved in fear expression. In either case, BNST-mediated modulation of contextual fear might summate with fear to the extinguished CS to yield reinstatement.

In conclusion, the present results reveal that distinct neural circuits mediate different forms of fear relapse. Here we show that the BNST is especially important for reinstatement, a form of relapse produced by the exposure of animals to aversive stimuli. Hence, selective manipulations of the BNST may be particularly effective in preventing fear relapse in aversive contexts. Ultimately, appreciating the circumstances that give rise to the return of fear will help in isolating circuit-specific therapies for combating fear relapse.

## Synthesis

The decision was a result of the Reviewing Editor Cheryl Sisk and the peer reviewers coming together and discussing their recommendations until a consensus was reached. A fact-based synthesis statement explaining their decision and outlining what is needed to prepare a revision is listed below. The following reviewer(s) agreed to reveal their identity: Dayan Knox

I have read your manuscript and the comments of the two reviewers and after consultation with each other, I ask that you revise your manuscript to address all of the specific points outlined in their reviews below. The additional methodological information and data (which you already have) requested by both reviewers will be important to include in your paper, and the issues raised by reviewer 2 on the relative "stressfulness" of fear reinstatement vs fear renewal should be addressed. The latter may require some reframing of the rationale and hypothesis for the study, and making the issue of whether reinstatement is more stressful than renewal a point of discussion.

### Reviewer 1

This is an interesting manuscript that replicates prior work showing that inactivation of the bed nucleus blocks reinstatement of extinguished fear behavior, and extending this work to show that bed nucleus activity does not seem to play a role in fear renewal. The manuscript was well-written and well-justified, and the experiments were conducted carefully. The authors are invited to comment on the following points, which I consider minor.

-The way the doses are reported is somewhat confusing. It initially looks like the authors infused 1 microliter per side (a volume that would cause concern), but that appears not to be the case upon further reading. This should be simplified and clarified.

-What did data look like in the animals with misplaced cannulas? Off-placement controls (at least for experiment 1) would enhance this work.

-It is unclear whether the end of the tract in figure 2 represents the end of the cannulas or the tip of the injector. Given that this particular example looks fairly ventral, some clarification to this point seems important.

-From figure 4, freezing seemed significantly higher at the start of the extinction than it was at the end of training. The authors are invited to comment on this observation.

-The symbols used in figure 4 are a too large, particular in B.

### Reviewer 2

Summary: The authors observed that inactivation of the BNST prior to testing fear reinstatement prevented the reinstatement of fear, while the same treatment had no effect on fear renewal or extinction memory recall.

Overall impression: The results of the study are interesting and the methods used to test the hypothesis are appropriate. There are some concerns.

Concern 1: It is difficult to determine why the authors concluded toward the end of the abstract that, "the BNST is critical for expressing context-shock associations that underlie stress-induced reinstatement, but not for contextual retrieval processes that underlie fear renewal." Isn't fear renewal stressful as well? The results only show that the BNST is critical for expression of fear reinstatement. Whether this is due to some stress phenomena remains unknown.

Concern 2: The interpretation that fear reinstatement is more, 'stressful', than fear renewal is interesting. The authors should note that both fear recovery paradigms result in similar amounts of fear recovery. This begs the question, 'why is fear reinstatement more stressful than fear renewal?' Without new data to address this question, there simply is not enough prior evidence to suggest that fear reinstatement is more stressful than fear renewal. Fear renewal depends on fear recovery from not being in the extinction context (this is stressful) and fear reinstatement depends on Context-US associations that prime fear memory retrieval (also stressful). The rationale and hypotheses for the study need to change. The hypothesis that the difference between the role of the BNST in fear reinstatement and fear renewal concerns stress differences between the two paradigms belongs in the Discussion.

Concern 3: It is interesting that reinstatement was observed irrespective of the context in which rats experienced the US. Does this suggest that the authors were observing some kind of generalized fear phenomena as opposed to fear reinstatement?

Concern 4: Authors should state how they scored freezing. Also, a qualitative description of what the data looked like for the misplacements (i.e. outside of the BNST) would be helpful and would help reinforced the assertion that the effects observed in this study are localized to the BNST.

Concern 5: For the Figure 2 legend, authors should put the magnification the image was taken at. Some more details about the microscope that was used would be helpful as well.

Concern 6: The authors should show data from the first extinction session as well as freezing during the reinstatement US presentation in Contexts A and B.

Concern 8: In the discussion the authors conclude that, "Hence, it is likely that BNST inactivation prevents reinstatement by reducing the expression of contextual fear, which is believed to be essential for the reinstatement effect (Bouton and Bolles, 1979; Westbrook et al., 2002; Bouton et al., 2006; Waddell et al., 2006)." However, the authors stated previously that reinstatement was equivalent whether rats were presented with the US in Context A or Context B. Doesn't this observation contradict the above statement, as it would appear contextual fear did not drive the reinstatement observed in this study?

## RESPONSE TO REVIEWERS

The authors thank the editor and the reviewers for their time and thoughtful comments. We have carefully revised our manuscript to address the concerns of the reviewers; we believe the paper is now greatly improved. Below, we elaborate on these changes in a point-by-point response.

Reviewer 1, General Comments: This is an interesting manuscript that replicates prior work showing that inactivation of the bed nucleus blocks reinstatement of extinguished fear behavior, and extending this work to show that bed nucleus activity does not seem to play a role in fear renewal. The manuscript was well written and well justified, and the experiments were conducted carefully. The authors are invited to comment on the following points, which I consider minor.

Reviewer 1, Concern 1: The way the doses are reported is somewhat confusing. It initially looks like the authors infused 1 microliter per side (a volume that would cause concern), but that appears not to be the case upon further reading. This should be simplified and clarified.

Authors' Response, Concern 1: We have clarified how the dosages are reported in the manuscript. All rats received a total volume of 0.3 µl per hemisphere (0.3 µl/min for 1 min). Muscimol was infused at a concentration of 1.0 µg/µl (i.e., 0.3 µg of muscimol per hemisphere) and NBQX was infused at a concentration of 10.0 µg/µl (i.e., 3.0 µg of NBQX per hemisphere).

Reviewer 1, Concern 2: What did data look like in the animals with misplaced cannulas? Off-placement controls (at least for experiment 1) would enhance this work.

Authors' Response, Concern 2: We have revised the manuscript to include data for 'off-target' infusion animals for Experiment 1. Animals with cannula placements that missed the BNST did not exhibit significant differences in fear reinstatement. We opted not to display these data in a figure, but the relevant statistics are now reported in the Results section.

Reviewer 1, Concern 3: It is unclear whether the end of the tract in figure 2 represents the end of the cannulas or the tip of the injector. Given that this particular example looks fairly ventral, some clarification to this point seems important.

Authors' Response, Concern 3: The original image for Figure 2 was selected because it is easy to see the entirety of both cannula tracts within the same slice. We have now replaced that image with a photomicrograph that depicts the termination of both injector tips in the same section (the new image for Figure 2 is from the same animal that was selected for the first submission of the manuscript). In this section, the tips of the injectors are located within the ventral portion of the BNST.

Reviewer 1, Concern 4: From figure 4, freezing seemed significantly higher at the start of the extinction than it was at the end of training. The authors are invited to comment on this observation.

Authors' Response, Concern 4: Freezing during the conditioning session is typically lower than that during the early trials of extinction because the USs experienced during conditioning elicit activity bursts that compete with freezing. Extinction occurs in the absence of shock, allowing freezing to be expressed without interruption by US-evoked activity bursts.

Reviewer 1, Concern 5: The symbols used in figure 4 are a too large, particular in B.

Authors' Response, Concern 5: To represent additional data from other phases of training (per Concern 6 of Reviewer 2), we have split Figure 4 into two new figures. Figure 4 now corresponds solely to the data collected from Experiment 1 (i.e., reinstatement), and Figure 5 now corresponds to the data obtained from Experiment 2 (i.e., renewal). Both figures have been rescaled to display the results more clearly.

Reviewer 2, General Comments: Summary: The authors observed that inactivation of the BNST prior to testing fear reinstatement prevented the reinstatement of fear, while the same treatment had no effect on fear renewal or extinction memory recall. Overall impression: The results of the study are interesting and the methods used to test the hypothesis are appropriate. There are some concerns.

Reviewer 2, Concern 1: It is difficult to determine why the authors concluded toward the end of the abstract that, "the BNST is critical for expressing context-shock associations that underlie stress-induced reinstatement, but not for contextual retrieval processes that underlie fear renewal." Isn't fear renewal stressful as well? The results only show that the BNST is critical for expression of fear reinstatement. Whether this is due to some stress phenomena remains unknown.

Authors' Response, Concern 1: These are important points (see also our response to Concern 2 below). Given the known role of the BNST in modulating stress responses (particularly in the presence of shock-associated contexts; see Sullivan et al., 2004, and Waddell et al., 2008), it is possible that BNST inactivation prevents reinstatement of fear by modulating stress activity leading up to onset of the CS (this possibility is addressed in part in the Discussion). However, we recognize that we have no independent index of 'stress' in our study. Accordingly, we have removed 'stress-induced' from the sentence in question. Our intent was not to suggest that renewal does not involve stress, rather that the mechanisms that trigger reinstatement vs. renewal may differentially depend on experiencing the CS in a shock-associated (stressful) context. That is, reinstatement requires an aversive 'trigger' to engage relapse (see Halladay et al., 2012), whereas simply encountering the extinguished CS outside of the extinction context (in a non-aversive setting) triggers renewal. BNST inactivation may prevent reinstatement by directly dampening context fear and accompanying stress hormone release associated with placement in a shock-associated context prior to CS onset.

Reviewer 2, Concern 2: The interpretation that fear reinstatement is more, 'stressful', than fear renewal is interesting. The authors should note that both fear recovery paradigms result in similar amounts of fear recovery. This begs the question, 'why is fear reinstatement more stressful than fear renewal?' Without new data to address this question, there simply is not enough prior evidence to suggest that fear reinstatement is more stressful than fear renewal. Fear renewal depends on fear recovery from not being in the extinction context (this is stressful) and fear reinstatement depends on Context-US associations that prime fear memory retrieval (also stressful). The rationale and hypotheses for the study need to change. The hypothesis that the difference between the role of the BNST in fear reinstatement and fear renewal concerns stress differences between the two paradigms belongs in the Discussion.

Authors' Response, Concern 2: Stress likely enhances renewal (see Knox et al., 2012), but renewal does not appear to require that the context itself is stressful. Indeed, in the renewal procedure, the test context is familiar and does not itself induce fear. In contrast, the reinstatement context has been paired with shock and generated high levels of fear (before CS onset; see also our response to Concern 3 below). This distinction we believe is critical for explaining why the BNST appears necessary for the manifestation of reinstatement but not renewal. We did not mean to suggest that reinstatement is more stressful than renewal per se (given the similar levels of relapse, as the reviewer mentioned), but rather that the mechanism that triggers these forms of relapse depend on experiencing the CS in an aversive context (reinstatement) or in a safe context (renewal) in which the CS has never been encountered. We have clarified these points in the manuscript. Also, we now include a statement indicating that the extent of relapse was similar between experiments.

Reviewer 2, Concern 3: It is interesting that reinstatement was observed irrespective of the context in which rats experienced the US. Does this suggest that the authors were observing some kind of generalized fear phenomena as opposed to fear reinstatement?

Authors' Response, Concern 3: We agree that this is an interesting observation (see also our response to Concern 7 below). Importantly, Westbrook et al. (2002) have demonstrated this phenomenon previously. Indeed, these and other findings (e.g., Morris et al., 2005a, 2005b; Goode et al., 2015) indicate that reinstatement to an extinguished CS can also occur outside of shock-associated contexts. That said, Westbrook et al. (2002) showed that reinstatement was limited to conditions in which rats experienced shocks in either the extinction or test contexts; footshocks outside of these contexts did not engage robust reinstatement. Accordingly, these data suggest that reinstatement by footshock relies on more than just fear generalization processes.

Reviewer 2, Concern 4: Authors should state how they scored freezing. Also, a qualitative description of what the data looked like for the misplacements (i.e. outside of the BNST) would be helpful and would help reinforced the assertion that the effects observed in this study are localized to the BNST.

Authors' Response, Concern 4: Within the Methods, we have elaborated on how we derived measures of freezing from locomotor activity. Also, as stated in our response to Concern 2 of Reviewer 1, we have now included the relevant statistics that describe testing data for off-target infusions in Experiment 1. At large, off-target vehicle- and drug-treated animals did not significantly differ at test, suggesting that our effects on reinstatement are specific to inactivation of BNST nuclei.

Reviewer 2, Concern 5: For the Figure 2 legend, authors should put the magnification the image was taken at. Some more details about the microscope that was used would be helpful as well.

Authors' Response, Concern 5: The image was collected at 10× using a Leica MZFLIII microscope. We have included these details within the Methods of the manuscript.

Reviewer 2, Concern 6: The authors should show data from the first extinction session as well as freezing during the reinstatement US presentation in Contexts A and B.

Authors' Response, Concern 6: To better accommodate these data, we have opted to split the original Figure 4 into two new figures (see also our response to Concern 5 of Reviewer 1). Now, Figure 4 corresponds to and shows the data from the reinstatement experiment, while Figure 5 depicts data from the renewal experiment.

Reviewer 2, Concern 7: In the discussion the authors conclude that, "Hence, it is likely that BNST inactivation prevents reinstatement by reducing the expression of contextual fear, which is believed to be essential for the reinstatement effect (Bouton and Bolles, 1979; Westbrook et al., 2002; Bouton et al., 2006; Waddell et al., 2006)." However, the authors stated previously that reinstatement was equivalent whether rats were presented with the US in Context A or Context B. Doesn't this observation contradict the above statement, as it would appear contextual fear did not drive the reinstatement observed in this study?

Authors' Response, Concern 7: Rats did not significantly differ at baseline based on whether they were shocked in Context A or B in our study, suggesting that rats shocked in A generalized their contextual fear of Context A to B (see also our response Concern 3 above). We have made additional changes to the manuscript to clarify these points.
